# Propofol suppresses cell viability, cell cycle progression and motility and induces cell apoptosis of ovarian cancer cells through suppressing MEK/ERK signaling via targeting circVPS13C/miR-145 axis

**DOI:** 10.1186/s13048-021-00775-3

**Published:** 2021-02-09

**Authors:** Huan Lu, Guanlin Zheng, Xiang Gao, Chanjuan Chen, Min Zhou, Longxin Zhang

**Affiliations:** Department of Anesthesiology, Fujian Provincial Maternity and Children’s Hospital, Affiliated Hospital of Fujian Medical University, No.18 daoshan Road, Fuzhou City, 350001 Fujian Province China

**Keywords:** Ovarian cancer, Propofol, circVPS13C, miR-145, MEK/ERK signaling

## Abstract

**Background:**

Propofol is a kind of common intravenous anaesthetic agent that plays an anti-tumor role in a variety of cancers, including ovarian cancer. However, the working mechanism of Propofol in ovarian cancer needs further exploration.

**Methods:**

The viability and metastasis of ovarian cancer cells were assessed by 3-(4,5-Dimethylthiazol-2-yl)-2,5-diphenyltetrazolium bromide (MTT) assay and transwell assays. Flow cytometry was used to evaluate the cell cycle and apoptosis. Quantitative real-time polymerase chain reaction (qRT-PCR) was used to examine the abundance of circular RNA vacuolar protein sorting 13 homolog C (circVPS13C) and microRNA-145 (miR-145). The target relationship between miR-145 and circVPS13C was predicted by circinteractome database and verified by dual-luciferase reporter assay, RNA-binding protein immunoprecipitation (RIP) assay and RNA-pull down assay. Western blot assay was used to detect the levels of phosphorylated extracellular regulated MAP kinase (p-ERK), ERK, p-MAP kinse-ERK kinase (p-MEK) and MEK, in ovarian cancer cells.

**Results:**

Propofol treatment suppressed the viability, cell cycle and motility and elevated the apoptosis rate of ovarian cancer cells. Propofol up-regulated miR-145 in a dose-dependent manner. Propofol exerted an anti-tumor role partly through up-regulating miR-145. MiR-145 was a direct target of circVPS13C. Propofol suppressed the progression of ovarian cancer through up-regulating miR-145 via suppressing circVPS13C. Propofol functioned through circVPS13C/miR-145/MEK/ERK signaling in ovarian cancer cells.

**Conclusion:**

Propofol suppressed the proliferation, cell cycle, migration and invasion and induced the apoptosis of ovarian cancer cells through circVPS13C/miR-145/MEK/ERK signaling in vitro.

## Highlights

1. Propofol hampers the proliferation, cell cycle and metastasis and enhances the apoptosis of ovarian cancer cells.
2. Propofol up-regulates miR-145 while down-regulates circVPS13C in ovarian cancer cells.
3. MiR-145 is a direct target of circVPS13C.
4. Propofol suppresses the development of ovarian cancer through suppressing MEK/ERK signaling via circVPS13C/miR-145 axis.

## Introduction

Ovarian cancer is a common gynecological cancer with the highest mortality rate among all kinds of gynecological cancers [5]. The 5-year survival rate of ovarian cancer patients remains low due to the difficulties in diagnosis at early stage. The combined therapy of surgery and chemotherapy is the standard therapy for ovarian cancer [6, 12]. However, chemoresistance is a big obstacle for ovarian cancer therapy. Thus, disclosing novel therapeutic targets is crucial to improve the prognosis of ovarian cancer patients.

Propofol is a kind of central nervous system anesthetic that is commonly used in surgical operations. The anti-tumor role of Propofol has been found in cancers [29, 31, 32]. For instance, Yang et al. found that Propofol suppressed the proliferation and viability of gastric cancer cells through up-regulating ING3 [29]. Besides, Propofol has been found to impede the invasion and induce the apoptosis of ovarian cancer cells [27]. Nevertheless, the underlying mechanism behind the function of Propofol in ovarian cancer cells is barely known.

Emerging articles have suggested that circular RNAs (circRNAs) could act as pivotal regulators in the pathology of many cancers [9, 26]. The dysregulation of circRNAs has been found in a variety of cancers, containing breast cancer, gastric cancer and colorectal cancer [35]. Bao et al. reported that circRNA vacuolar protein sorting 13 homolog C (circVPS13C) was up-regulated in ovarian cancer, and circVPS13C accelerated the progression of ovarian cancer [1]. However, the working mechanism of circVPS13C in ovarian cancer remains to be revealed.

MicroRNAs (miRNAs) are small non-coding RNAs (ncRNAs) with 21–23 nucleotides. MiRNAs could regulate gene expression through directly targeting corresponding messenger RNAs (mRNAs) via their miRNA binding sites in mRNAs [13, 17]. MiR-145 played an anti-tumor role in many cancers. For instance, Sui et al. found that Lidocaine suppressed the malignant behaviors of gastric cancer cells through up-regulating miR-145 [22]. Ding et al. claimed that miR-145 restrained the development of breast cancer via TGF-β1 [3]. As for ovarian cancer, Zhu et al. found that miR-145 elevated the sensitivity of ovarian cancer cells to paclitaxel via Sp1 and Cdk6 [36]. However, the role of miR-145 in Propofol-mediated influence of ovarian cancer cells remains to be uncovered.

We found that Propofol inhibited the viability, cell cycle and metastasis while induced the apoptosis of ovarian cancer cells. CircVPS13C/miR-145 axis was identified for the first time, and this signal pathway provided novel insight of the working mechanism of Propofol in ovarian cancer cells.

## Materials and methods

### Clinical tissue samples

Forty pairs of ovarian cancer tissue samples and adjacent non-tumor tissue samples were collected from patients diagnosed with ovarian cancer at Fujian Provincial Maternity and Children’s Hospital. Written informed consents have been provided by all subjects before the surgery. This experiment was authorized by the Institutional Ethics Committee of Fujian Provincial Maternity and Children’s Hospital.

### Cell culture

Human normal ovarian epithelial cell line IOSE-80, two ovarian cancer cell lines (A2780 and SKOV3) and human embryonic kidney cell line 293 T were purchased from BeNa Culture Collection (Beijing, China) and maintained in Dulbecco’s Modified Eagle Medium (DMEM) added with 10% fetal bovine serum (FBS), 100 U/mL penicillin, and 100 mg/mL streptomycin at 37 °C incubator with 5% CO_2_.

### Propofol treatment

The blood concentration of Propofol in clinical usage is 1 μg/mL-10 μg/mL. Ovarian cancer cells treated with 3 μg/mL, 6 μg/mL, 9 μg/mL Propofol or dimethyl sulfoxide (DMSO, Sigma, St. Louis, MO, USA) were utilized for further analysis.

### 3-(4,5-Dimethylthiazol-2-yl)-2,5-diphenyltetrazolium bromide (MTT) assay

Cell Proliferation Reagent Kit (Roche, Shanghai, China) was used to examine the viability of ovarian cancer cells. After indicated treatment, MTT reagent (20 μL/5 mg/mL) was added to the wells of 96-well plates. The formazan products were dissolved using 200 μL DMSO. The spectrophotometric absorbance was detected at 490 nm.

### Flow cytometry

For cell cycle analysis, the treated or untreated ovarian cancer cells were collected and rinsed using phosphate buffered saline (PBS) followed by immobilization in 70% ethanol overnight at − 20 °C. After RNase (Sigma) digestion, DNA content was dyed using 20 mg/mL propidium iodide (PI; Sigma). Cell cycle of ovarian cancer cells was analyzed using the flow cytometer.

For apoptosis analysis, ovarian cancer cells after Propofol exposure for 72 h were double-stained with fluorescein isothiocyanate (FITC)-Annexin V (BD Biosciences, Franklin Lakes, NJ, USA) and PI (BD Biosciences). The normal ovarian cancer cells were distinguished from necrotic, early apoptotic and late apoptotic ovarian cancer cells using the flow cytometer.

### Transwell assays

The abilities of migration and invasion in ovarian cancer cells were assessed by transwell assays. To assess the invasion ability, upper chambers were pre-coated with Matrigel (BD Biosciences). Ovarian cancer cells after Propofol treatment for 24 h were suspended in serum-free medium. 100 μL cell suspension was plated in the upper chambers (Costar, Corning, NY, USA). 500 μL culture medium added with 10% FBS was added to the lower chambers. The invaded cells were stained with crystal violet and counted after 24 h-incubation. To assess the migration ability, cell suspension was plated in un-coated upper chambers, and the other steps were similar as transwell invasion assay.

### Quantitative real-time polymerase chain reaction (qRT-PCR)

RNA samples were isolated using TRIzol reagent (Invitrogen, Carlsbad, CA, USA). For circVPS13C reverse transcription, 1.0 μg RNA was used to synthesize complementary DNA (cDNA) with a reverse transcription kit (Qiagen, Hilden, Germany). For the reverse transcription of miRNA, One step miRNA RT Kit (Haigene, Harbin, China) was used. Divergent primers were used to conduct PCR reaction on Rotorgene 6000 series PCR machine (Qiagen). The relative expression of circVPS13C and miR-145 was normalized to internal controls (U6 and glyceraldehyde-3-phosphate dehydrogenase (GAPDH)) with the 2^−ΔΔCt^ formula, respectively. The divergent primers were displayed in Table 1.

**Table 1:** Primer sequences in qRT-PCR assay

Gene	Primer
circVPS13C	TATAATTTTGTCTGCTTCATTTA (forward; F) TTAACACAGTCTAAAGTCTCAGAA (reverse; R)
miR-145	GTCCAGTTTTCCCAGGAATCCCT (F) AGGGATTCCTGGGAAAACTGGAC (R)
U6	CTCGCTTCGGCAGCACA (F) AACGCTTCACGAATTTGCGT (R)
GAPDH	AGAAGGCTGGGGCTCATTTG (F) AGGGGCCATCCACAGTCTTC (R)

### Cell transfection

MiR-145 mimics (miR-145), miRNA negative control (miR-NC), miR-145 inhibitor (anti-miR-145), anti-NC, circVPS13C small interfering RNA (si-circVPS13C), si-NC, circVPS13C overexpression plasmid (oe-VPS13C) and vector were obtained from Genepharma (Shanghai, China).

### Dual-luciferase reporter assay

The interaction between miR-145 and circVPS13C was predicted by circinteractome database. The sequences of circVPS13C with the complementary sites of miR-145, including the wild-type sequence (WT), Position 89–95 mutant sequence (MUT1), Position 272–278 mutant sequence (MUT2) or double mutant sequence (MUT1 + 2), were amplified and inserted into pGL3 vectors (Promega, Madison, WI, USA) to obtain reporter plasmids (WT, MUT1 (89–95), MUT2 (272–278) and MUT1 + 2). 293 T cells were co-transfected with these reporter plasmids and miR-NC or miR-145, and the luciferase activity in each group was analyzed using luciferase assay kit (Promega) after transfection for 48 h.

### RNA-binding protein immunoprecipitation (RIP) assay

Ovarian cancer cells were disrupted using RIP buffer (Millipore, Bedford, MA, USA), cell lysates were then incubated with protein-A/G Sepharose beads (Bio-Rad, Hercules, CA, USA) pre-coated with 3 μg Argonaute-2 (Ago2) antibody or control Immunoglobulin G (IgG) antibody for 3 h. The RNA complexes were isolated using TRIzol reagent (Invitrogen) and examined by qRT-PCR.

### RNA-pull down assay

MiR-145 and miR-NC were biotinylated to generate bio-miR-145 and bio-miR-NC. 2 μg cell lysates were incubated with 100 pmol bio-miR-145 or bio-miR-NC. Beads were washed for three times followed by detection the expression of circVPS13C using qRT-PCR.

### Western blot assay

Ovarian cancer cells were disrupted using cell lysis buffer (Promega) on ice for 30 min and centrifuged at 12000 rpm for 30 min. The supernatant was transferred into the new centrifuge tube, and the concentration of protein samples was detected using the BCA-200 Protein Assay kit (Pierce, Rockford, IL, USA). Protein samples were separated by 10% sodium dodecyl sulfate polyacrylamide gel electrophoresis (SDS-PAGE) gel and transferred to the polyvinylidene fluoride (PVDF) membrane (Millipore). The non-specific sites in the membrane were blocked using 5% skim milk for 1 h, followed by incubation with primary antibodies and horseradish peroxidase (HRP)-labeled secondary antibody. The blots were visualized using the enhanced chemiluminescent (ECL) system (Beyotime, Shanghai, China). The primary antibodies, including phosphorylated extracellular regulated MAP kinase (p-ERK; ab214036), ERK (ab17942), p-MAP kinse-ERK kinase (p-MEK; ab96379), MEK (ab178876) and GAPDH (ab181602) were purchased from Abcam (Cambridge, MA, USA).

### Statistical analysis

All experiments were repeated for at least three times, and the data were analyzed using GraphPad Prism 7.0 and displayed as mean ± standard deviation (SD). The differences were evaluated using Student’s *t*-test or one-way analysis of variance (ANOVA) followed by Tukey’s post hoc test. Differences were considered as statistically significant when *P* < 0.05.

## Results

### Propofol inhibits the viability, cell cycle and metastasis while triggers the apoptosis of ovarian cancer cells

**Fig. 1:**
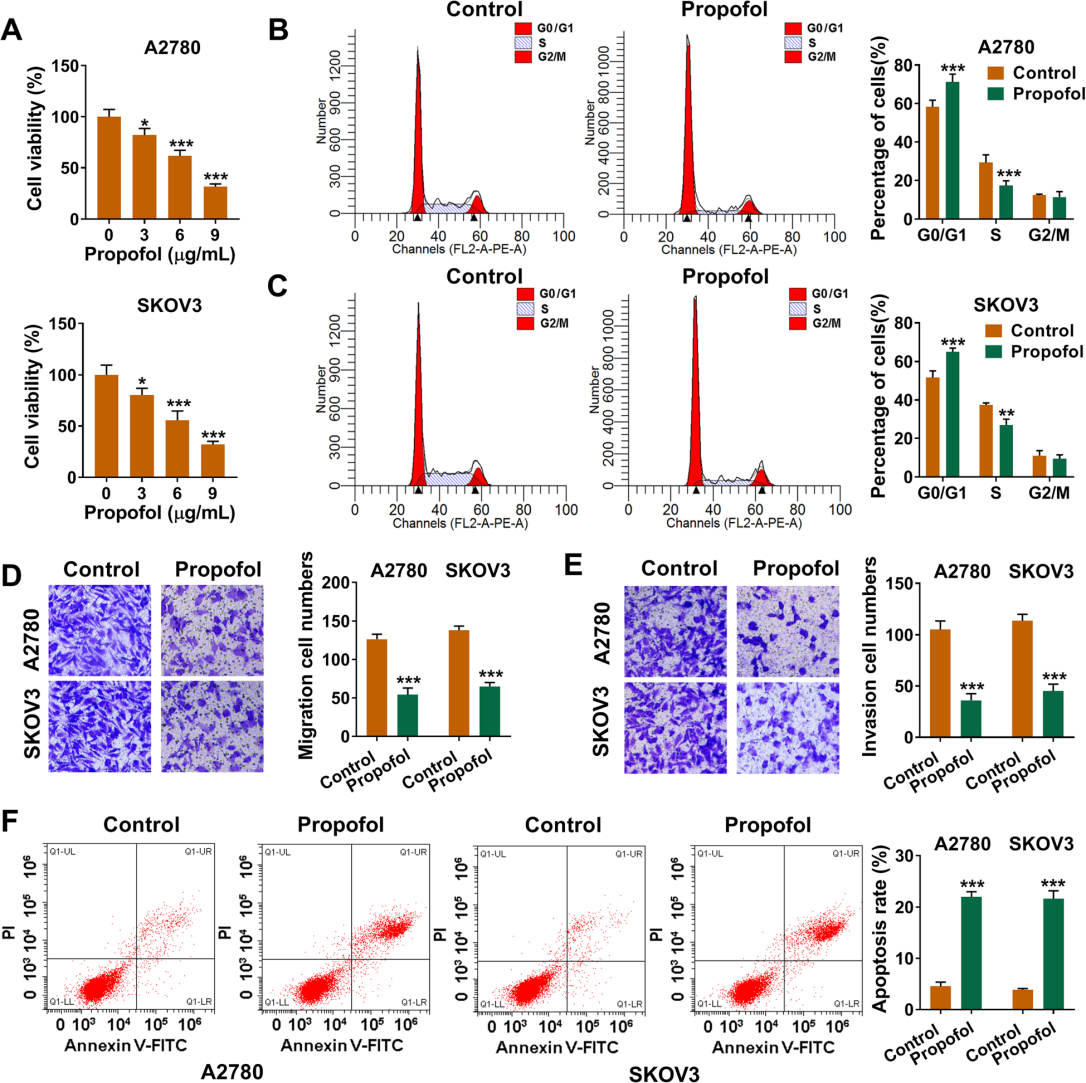
Propofol inhibits the viability, cell cycle and metastasis while triggers the apoptosis of ovarian cancer cells. **a** The cell viability of A2780 and SKOV3 cells exposed to different concentrations of Propofol (3 μg/mL, 6 μg/mL or 9 μg/mL) for 48 h was analyzed by MTT assay. **b**-**f** A2780 and SKOV3 cells were divided into two groups: Control group and Propofol treatment group (6 μg/mL). **b** and **c** The percentages of ovarian cancer cells in G0/G1, S and G2/M were measured by flow cytometry. **d** and **e** Transwell assays were used to examine the abilities of migration and invasion of ovarian cancer cells (magnification: 100 ×). The ruler in figures indicates 100 μM. **f** The apoptosis rate (early and late apoptosis) of ovarian cancer cells was analyzed by flow cytometry. *P < 0.05, **P < 0.01, ***P < 0.001

A2780 and SKOV3 cells were treated with 3 μg/mL, 6 μg/mL or 9 μg/mL Propofol for 48 h, and cell viability was analyzed by MTT assay. As existed in Fig. 1a, cell viability was dose-dependently decreased with the increased concentration of Propofol. Meanwhile, the influence of Propofol on the cell cycle, migration, invasion and apoptosis of ovarian cancer cells was further explored. After treating with 6 μg/mL Propofol for 48 h, the cell cycle was inhibited at G1/S transition (Fig. 1b and c). Propofol exposure also suppressed the migration and invasion of ovarian cancer cells (magnification: 100 ×; Fig. 1d and e). Besides, after Propofol treatment, the apoptosis rate (early and late stage apoptosis) was significantly elevated (Fig. 1f). In summary, Propofol suppressed the malignant potential of ovarian cancer cells in vitro.

### Propofol up-regulates the level of miR-145 in ovarian cancer cells

**Fig. 2:**
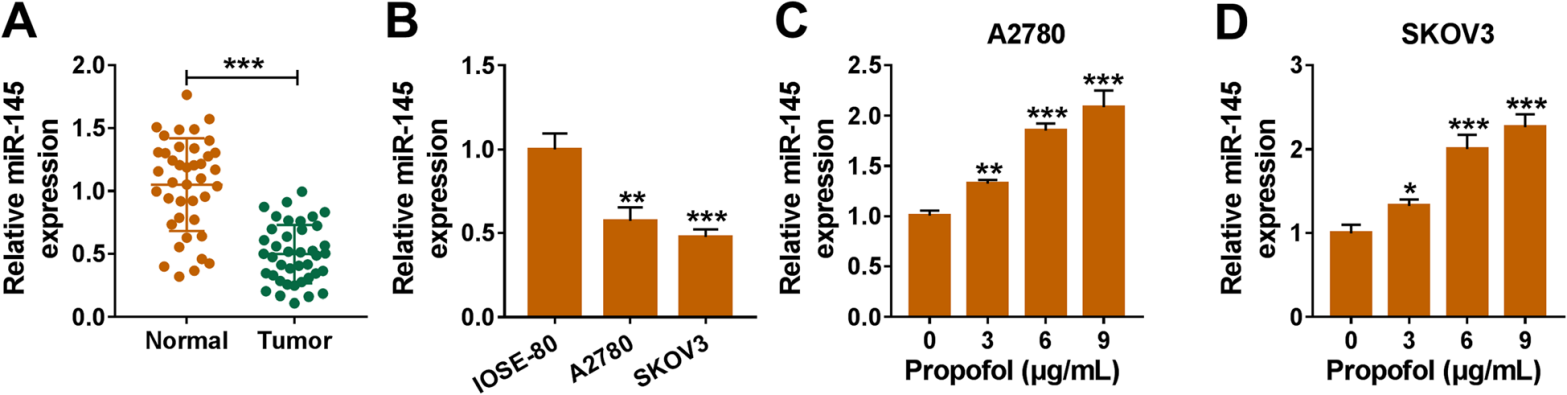
Propofol up-regulates the level of miR-145 in ovarian cancer cells. **a** qRT-PCR was employed to detect the expression of miR-145 in ovarian cancer samples and non-tumor samples. **b** miR-145 level in human normal ovarian epithelial cell line IOSE-80 and two ovarian cancer cell lines was detected by qRT-PCR. **c** and **d** A2780 and SKOV3 cells were treated with increased doses of Propofol, and the expression of miR-145 was detected by qRT-PCR. *P < 0.05, **P < 0.01, ***P < 0.001

MiR-145 level was notably reduced in ovarian cancer tissues compared with adjacent normal tissues (Fig. 2a). Also, there was a significant decrease in miR-145 level in ovarian cancer cells than that in IOSE-80 cells (Fig. 2b). After treating with increased doses of Propofol for 48 h, the level of miR-145 was enhanced in a dose-dependent manner (Fig. 2c and d). These results revealed that Propofol enhanced miR-145 level in ovarian cancer cells.

### Propofol-induced damage in ovarian cancer cells is attenuated by the addition of anti-miR-145

**Fig. 3:**
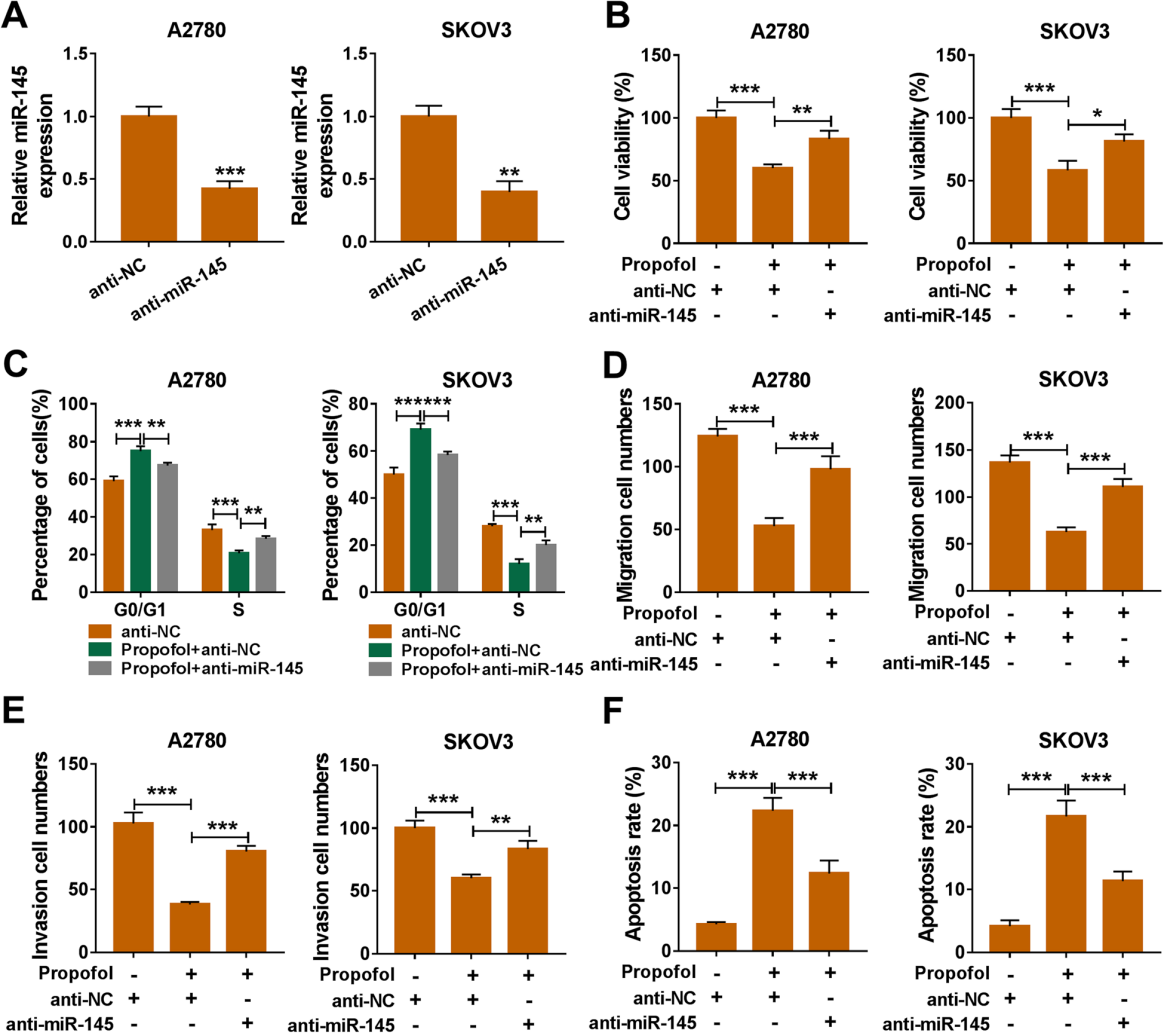
Propofol-induced damage in ovarian cancer cells is attenuated by the addition of anti-miR-145. **a** After transfecting with anti-NC or anti-miR-145, the expression of miR-145 was detected by qRT-PCR. (B-F) Ovarian cancer cells were divided into three groups, including anti-NC group, Propofol + anti-NC group, Propofol + anti-miR-145 group. **b** MTT assay was applied to examine the viability of ovarian cancer cells in different groups. **c** The influence of Propofol and anti-miR-145 in the cell cycle of ovarian cancer cells was analyzed by flow cytometry. **d** and **e** The migration ability and invasion ability of ovarian cancer cells were assessed by transwell assays. **f** The apoptosis rate in different treatment groups was analyzed by flow cytometry. *P < 0.05, **P < 0.01, ***P < 0.001

The interference efficiency of miR-145 inhibitor (anti-miR-145) was assessed by qRT-PCR. As shown in Fig. 3a, anti-miR-145 transfection notably decreased the level of miR-145 in ovarian cancer cells. Propofol-mediated suppressive impact on the viability of ovarian cancer cells was partly attenuated by the transfection of anti-miR-145 (Fig. 3b). The cell cycle was arrested with Propofol treatment, and the interference of miR-145 recovered the cell cycle of ovarian cancer cells (Fig. 3c). Propofol-mediated inhibition on the migration and invasion was counteracted by the addition of anti-miR-145 (Fig. 3d and e). Meanwhile, the apoptosis rate was decreased by the introduction of anti-miR-145 that was increased by the treatment of Propofol (Fig. 3f). These findings suggested that Propofol-mediated injury could be partly alleviated by the transfection of anti-miR-145 in ovarian cancer cells.

### CircVPS13C directly interacts with miR-145

**Fig. 4:**
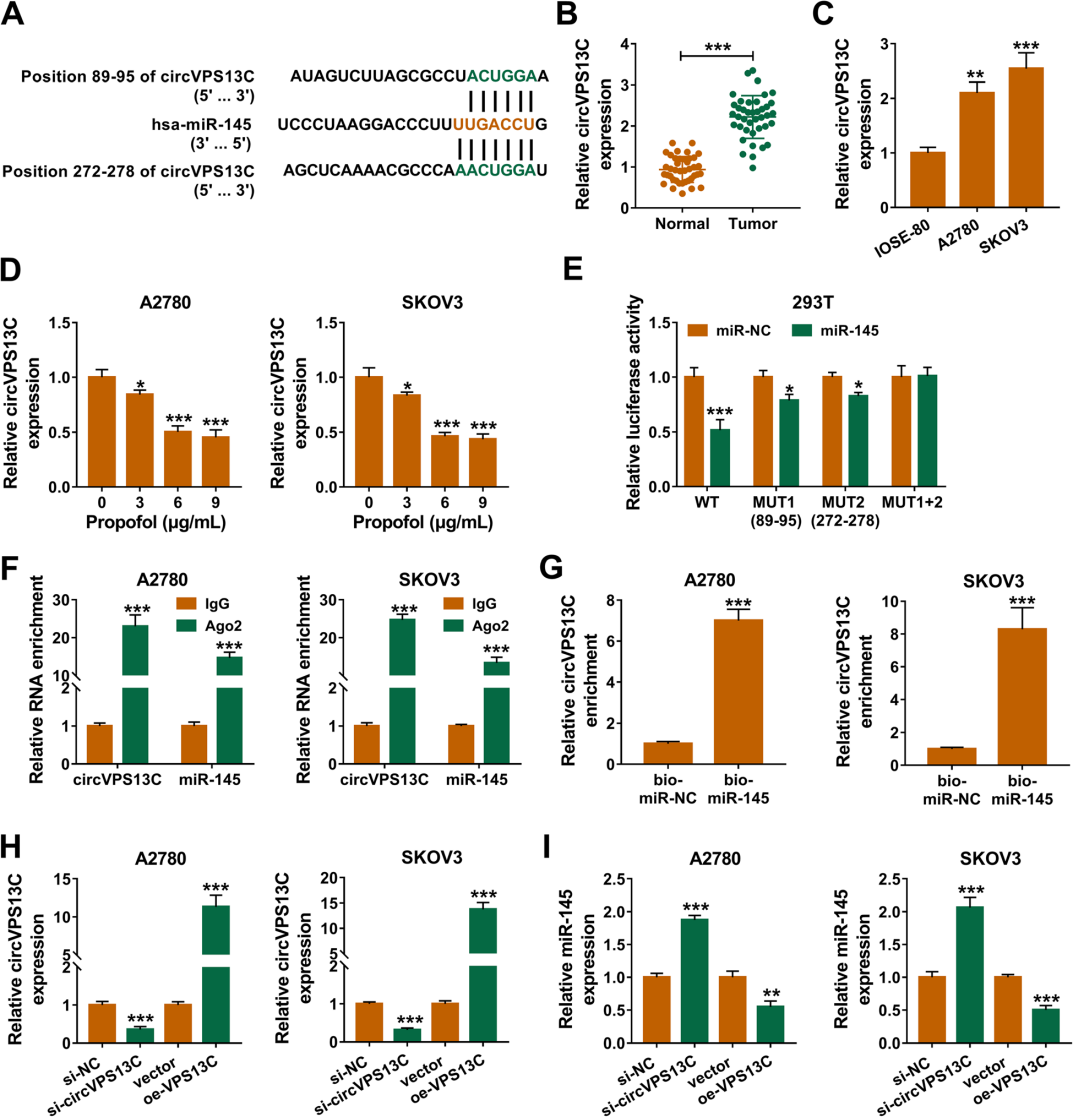
CircVPS13C directly interacts with miR-145. **a** There existed two sites in circVPS13C that were complementary with miR-145 (predicted by circinteractome database), including position 89–95 and position 272–278. **b** and **c** The abundance of circVPS13C in adjacent normal tissues, ovarian cancer tissues, IOSE-80 cell line and two ovarian cancer cell lines was detected by qRT-PCR. **d** Ovarian cancer cells were exposed to different concentrations of Propofol, and the level of circVPS13C was detected by qRT-PCR. **e** The wild-type sequence of circVPS13C that contains the two complementary sites with miR-145, the single mutant sequence (89–95) of circVPS13C, the single mutant sequence (272–278) of circVPS13C and the double mutant sequence of circVPS13C were amplified and cloned into luciferase reporter vectors, generating WT, MUT1 (89–95), MUT2 (272–278) and MUT1 + 2, respectively. Dual-luciferase reporter assay was used to test which position in circVPS13C could directly bind to miR-145, and the luciferase activity was detected in 293 T cells co-transfected with these reporter plasmids and miR-NC or miR-145. **f** RIP assay was used to test whether there existed spatial interaction between miR-145 and circVPS13C in ovarian cancer cells. **g** The interaction between miR-145 and circVPS13C was tested by RNA-pull down assay. **h** and **i** Ovarian cancer cells were transfected with si-NC, si-circVPS13C, vector or oe-circVPS13C. The levels of circVPS13C and miR-145 in transfected ovarian cancer cells were examined by qRT-PCR. *P < 0.05, **P < 0.01, ***P < 0.001

Through using circinteractome software, miR-145 was found as a potential target of circVPS13C (Fig. 4a). There were two positions (Position 89–95 and Position 272–278) in circVPS13C that were complementary with miR-145 sequence (Fig. 4a). CircVPS13C level was higher in ovarian cancer tissues in contrast to that in adjacent normal tissues (Fig. 4b). Also, the level of circVPS13C was notably up-regulated in ovarian cancer cells than that in IOSE-80 cells (Fig. 4c). Propofol treatment reduced the expression of circVPS13C in a dose-dependent manner (Fig. 4d). We wondered which position in circVPS13C could bind to miR-145, and wild-type sequence, single mutation (MUT1 and MUT2) or double mutation of circVPS13C were amplified and cloned into luciferase reporter vectors, termed as WT, MUT1 (89–95), MUT2 (272–278) and MUT1 + 2. 293 T cells were co-transfected with miR-NC or miR-145 and these reporter vectors. As presented in Fig. 4e, the luciferase activity was decreased in WT, MUT1 and MUT2 group when co-transfected with miR-145 rather than miR-NC. Among these groups, the decrease in luciferase activity was the most obvious in WT and miR-145 co-transfected group than that in WT and miR-NC co-transfected group (Fig. 4e), suggested that both these two sites in circVPS13C could directly bind to miR-145. The results of RIP assay showed that both circVPS13C and miR-145 were substantially enriched in Ago2 group (Fig. 4f), suggested that both these two genes could bind to Ago2-contained RNA inducing silence complex (RISC). The results of RNA-pull down assay revealed that circVPS13C was enriched when using biotinylated miR-145 (bio-miR-145, Fig. 4g), suggested that there existed spatial interaction between miR-145 and circVPS13C. qRT-PCR was applied to uncover the regulatory relationship between miR-145 and circVPS13C. The transfection efficiencies of si-circVPS13C and oe-circVPS13C were high in ovarian cancer cells (Fig. 4h). CircVPS13C knockdown elevated the level of miR-145, and the accumulation of circVPS13C decreased the level of miR-145 (Fig. 4i). Collectively, circVPS13C directly interacted with and down-regulated miR-145.

### Propofol inhibits the progression of ovarian cancer via circVPS13C/miR-145 axis

**Fig. 5:**
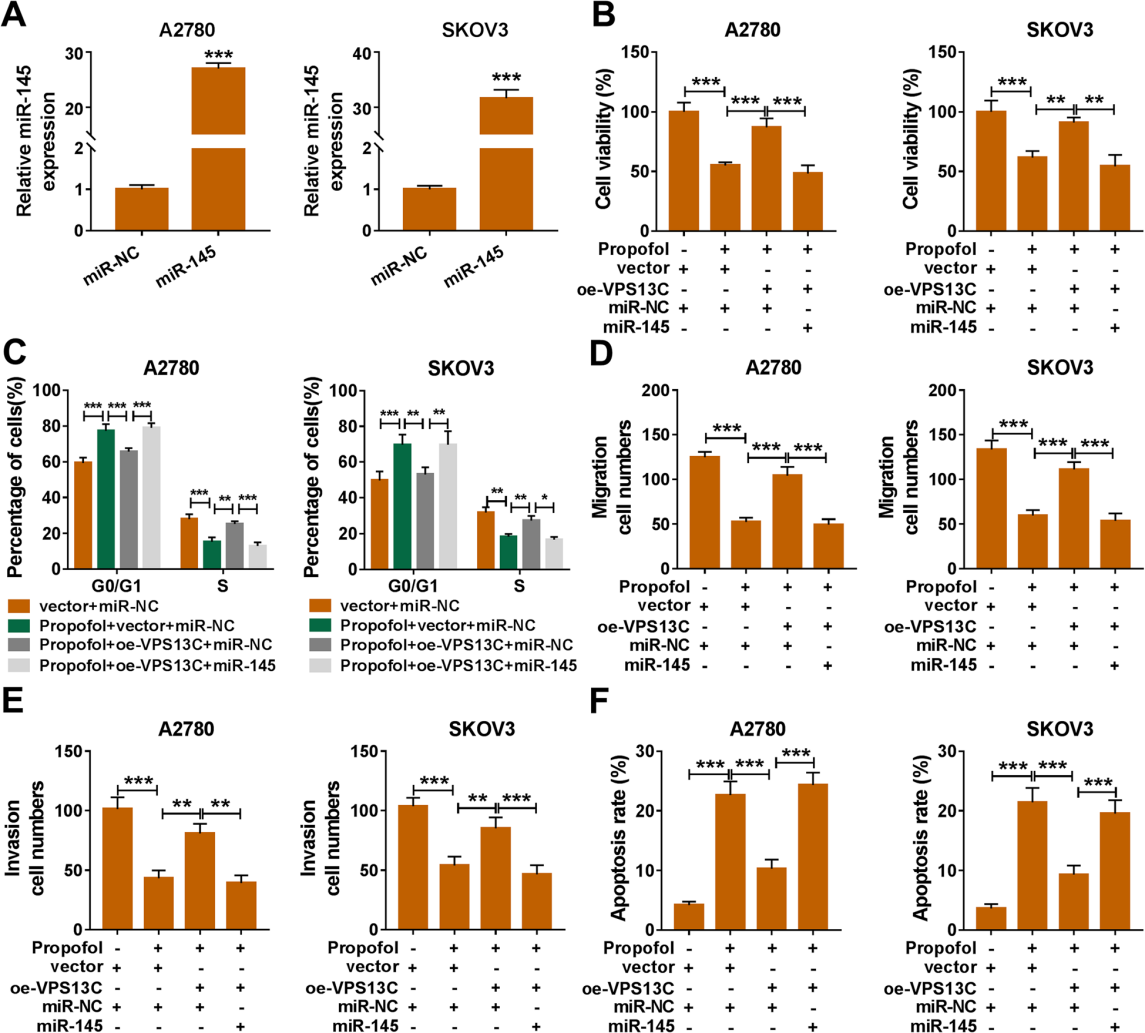
Propofol inhibits the progression of ovarian cancer via circVPS13C/miR-145 axis. **a** We transfected miR-145 or miR-NC into ovarian cancer cells, and the level of miR-145 was analyzed by qRT-PCR. (B-F) Ovarian cancer cells were divided into four groups, including vector + miR-NC group, Propofol + vector + miR-NC group, Propofol + oe-VPS13C + miR-NC group and Propofol + oe-VPS13C + miR-145 group. **b** Cell viability was assessed by MTT assay. **c** Cell cycle was measured by flow cytometry. **d** and **e** The migration and invasion capacities of ovarian cancer cells were assessed by transwell assays. **f** The apoptotic ovarian cancer cells were distinguished from normal or necrotic cells by flow cytometry. *P < 0.05, **P < 0.01, ***P < 0.001

The overexpression efficiency of miR-145 mimics (miR-145) was high in ovarian cancer cells (Fig. 5a). The accumulation of circVPS13C recovered the viability, cell cycle, migration and invasion of Propofol-induced ovarian cancer cells, and the addition of miR-145 suppressed the malignant behaviors of ovarian cancer cells again (Fig. 5b-e). CircVPS13C overexpression inhibited the apoptosis of Propofol-treated ovarian cancer cells, and the apoptosis rate was enhanced by the addition of miR-145 (Fig. 5f). Taken together, Propofol inhibited the proliferation, cell cycle and metastasis and promoted the apoptosis of ovarian cancer cells via circVPS13C/miR-145 axis.

### Propofol suppresses MEK/ERK signaling through circVPS13C/miR-145 axis in ovarian cancer cells

**Fig. 6:**
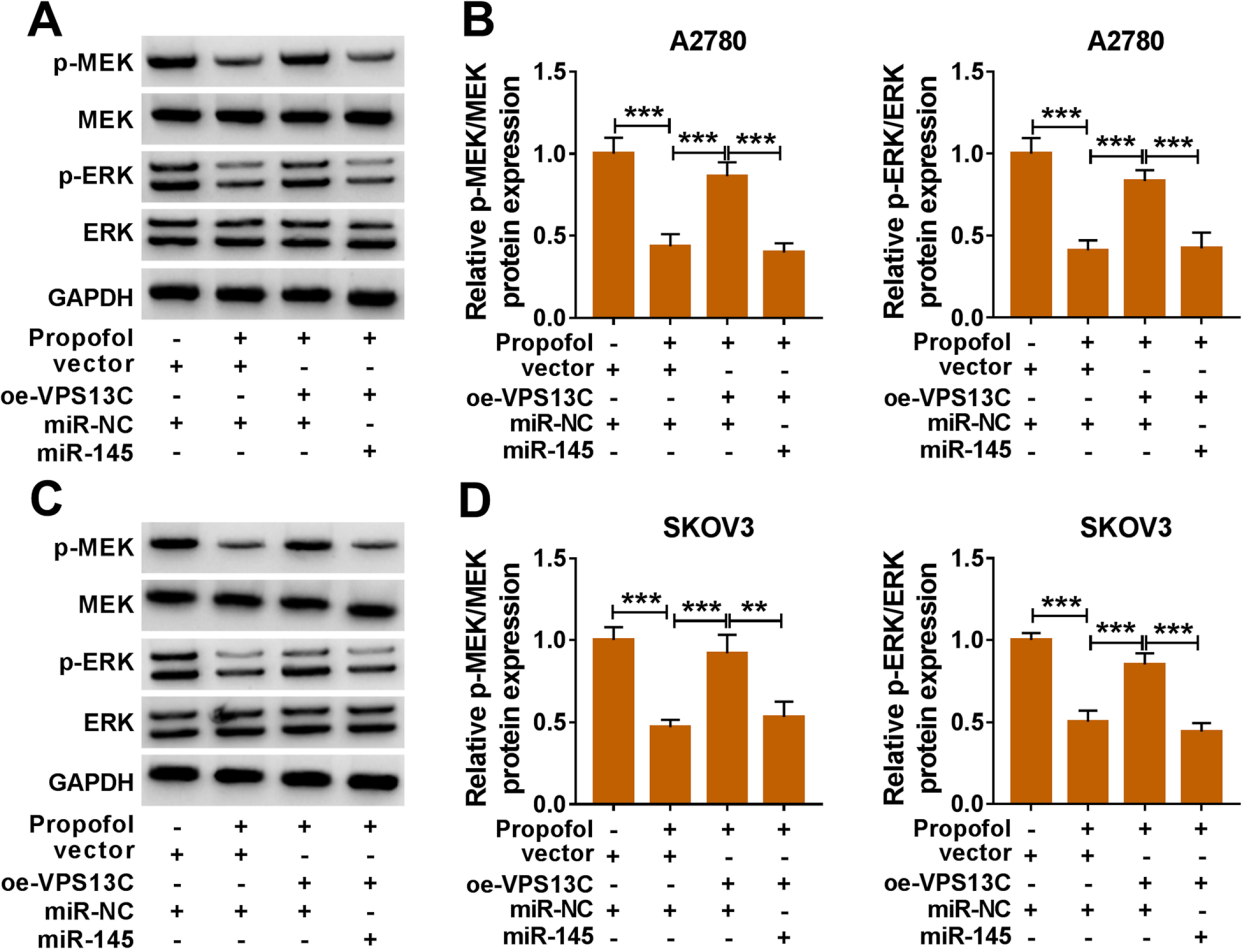
Propofol suppresses MEK/ERK signaling through circVPS13C/miR-145 axis in ovarian cancer cells. Ovarian cancer cells were divided into four groups, including vector + miR-NC group, Propofol + vector + miR-NC group, Propofol + oe-VPS13C + miR-NC group and Propofol + oe-VPS13C + miR-145 group. **a**-**d** The levels of p-MEK, MEK, p-ERK and ERK in ovarian cancer cells were measured by Western blot assay and quantified using Image J software. **P < 0.01, ***P < 0.001

Propofol treatment down-regulated the phosphorylation of MEK and ERK in A2780 and SKOV3 cells, and the addition of oe-VPS13C recovered the phosphorylation levels of MEK and ERK (Fig. 6a-d). Besides, the phosphorylation levels of MEK and ERK were reduced in Propofol + oe-VPS13C + miR-145 group compared with Propofol + oe-VPS13C + miR-NC group (Fig. 6a-d). These findings revealed that Propofol inhibited the progression of ovarian cancer through suppressing MEK/ERK signaling via circVPS13C/miR-145 axis.

## Discussion

Propofol is a common central nervous system anesthetic that is responsible for the induction and maintenance of anesthesia. Propofol played an anti-tumor role in a variety of cancers through suppressing the growth of tumors [2, 34], inducing the apoptosis of cancer cells [2, 20] and inhibiting the metastasis of cancer cells [27, 28]. The anti-tumor role of Propofol in ovarian cancer has also been reported before [11, 24, 27]. For instance, Huang et al. claimed that Propofol hampered the invasion and proliferation of ovarian cancer cells through modulating miR-9/NF-κB signaling [11]. Sun et al. found that Propofol blocked the proliferation and chemoresistance of ovarian cancer cells [24].

We found that Propofol treatment suppressed the viability, cell cycle, migration and invasion and promoted the apoptosis of ovarian cancer cells, which was in agreement with the findings of previous articles [11, 24, 27].

Previous articles have reported that Propofol exerted its functions through regulating miRNAs in cancers. For instance, Propofol impeded the proliferation, motility and accelerated the apoptosis of hepatocarcinoma cells through suppressing miR-374a [18]. Yu et al. found that Propofol suppressed the proliferation and motility of pancreatic cancer cells through miR-328/ADAM8 axis [33]. As for ovarian cancer, Su et al. found that Propofol facilitated the apoptosis of epithelial ovarian cancer cells through up-regulating miRNA let-7i [20]. MiR-145 functioned as a tumor suppressor in many types of cancers, including ovarian cancer. For instance, miR-145 blocked the metastasis of human colorectal cancer cells through regulating PAK4-dependent pathway [19]. Zhu et al. claimed that miR-145 enhanced the drug sensitivity of paclitaxel in ovarian cancer cells via Sp1 and Cdk6 [36]. We found that miR-145 was notably down-regulated in ovarian cancer tissues and cell lines compared with para-carcinoma tissues and normal ovarian epithelial cell line. Subsequently, we examined the effect of Propofol on the expression of miR-145 to illustrate the working mechanism of Propofol in ovarian cancer cells. The level of miR-145 was up-regulated with the increased concentrations of Propofol. Further experiments demonstrated that Propofol exerted an anti-tumor role through up-regulating miR-145 in ovarian cancer cells.

CircVPS13C was found to be up-regulated in ovarian cancer tissues and cell lines in contrast to that in adjacent non-tumor tissues and normal ovarian epithelial cell line, which was consistent with former article [1]. CircRNAs are involved in the initiation and development of cancers mainly through acting as miRNAs sponges [7, 14, 25]. For example, circABCB10 accelerated the progression of breast cancer via sponging miR-1271 [16]. CircITCH suppressed the development of bladder cancer through sponging miR-17/miR-224 [30]. Here, the direct interaction between miR-145 and circVPS13C was identified through conducting dual-luciferase reporter assay, RIP assay and RNA-pull down assay. Subsequently, we found the accumulation of circVPS13C partly reversed Propofol-mediated influence in ovarian cancer cells, and the addition of miR-145 suppressed the malignant potential of ovarian cancer cells again, suggested that Propofol suppressed the progression of ovarian cancer through regulating circVPS13C/miR-145 axis.

The ERK signaling pathway exhibits vital functions in regulating cellular biological behaviors, including cell viability, proliferation and apoptosis [4, 8]. Accumulating articles have pointed the important roles of ERK pathway in CRC progression. For instance, Sun et al. demonstrated that USP11 contributed to the proliferation ability and motility of CRC cells through up-regulating PPP1CA-mediated activation of ERK pathway [23]. Huang et al. found that BZW2 accelerated the malignant behaviors of CRC cells through activating ERK signaling [10]. Furthermore, the functional association between Propofol and the activity of ERK signaling has also been reported by former studies. Li et al. demonstrated that miR-34a silencing protected neuroblastoma cells from Propofol-induced neurotoxicity through regulating ERK signaling [15]. Su et al. found that Propofol restrained the proliferation ability and triggered the apoptosis of cardia cancer cells through in-activating ERK signaling [21]. In this study, the effect of Propofol/circVPS13C/miR-145 axis on the activation of MEK/ERK signaling was explored in ovarian cancer cells. The results revealed that Propofol treatment inhibited the activation of MEK/ERK signaling through up-regulating miR-145 via down-regulating circVPS13C.

In further study, the in vivo role of Propofol/circVPS13C/miR-145 axis in ovarian cancer tumor growth needs further exploration.

## Conclusion

In conclusion, our study provided a new insight that circVPS13C/miR-145 axis was involved in Propofol-mediated anti-tumor role in ovarian cancer. CircVPS13C/miR-145/MEK/ERK axis might be a promising therapeutic target for ovarian cancer.

## Data Availability

The analyzed data sets generated during the present study are available from the corresponding author on reasonable request.

## Abbreviations

MTT: Dimethylthiazol-2-yl)-2,5-diphenyltetrazolium bromide
qRT-PCR: Quantitative real-time polymerase chain reaction
circVPS13C: Circular RNA vacuolar protein sorting 13 homolog C
RIP: RNA-binding protein immunoprecipitation
DMEM: Dulbecco’s Modified Eagle Medium
ANOVA: Analysis of variance
SD: Standard deviation
